# TAS1R3 influences GTPase-dependent signaling in human islet β-cells

**DOI:** 10.3389/fendo.2025.1695980

**Published:** 2025-12-08

**Authors:** Rajakrishnan Veluthakal, Miwon Ahn, Eunjin Oh, Debbie C. Thurmond

**Affiliations:** Department of Molecular and Cellular Endocrinology, Arthur Riggs Diabetes and Metabolism Research Institute of City of Hope, Duarte, CA, United States

**Keywords:** GPCR, type 2 diabetes, human islets, G-protein, cAMP

## Abstract

**Background:**

The taste receptor type 1 member 3 (TAS1R3), a G protein-coupled receptor (GPCR), is expressed in pancreatic islet β-cells where it may influence intracellular signaling pathways critical for β-cell function. Although TAS1R3 is known to couple to heterotrimeric G-proteins, its potential influence on small GTPases—key regulators of vesicle trafficking, cytoskeletal remodeling, and signal transduction—remains unexplored. Investigating how TAS1R3 modulates small GTPase activity could uncover mechanisms by which β cells regulate insulin secretion and adapt to metabolic cues.

**Objective:**

We questioned whether activation of endogenous TAS1R3 in human islets or clonal human β-cells are necessary for glucose-stimulated insulin secretion *via* activation of small GTPases.

**Methods and results:**

We found that pharmacological TAS1R3 inhibition (lactisole) in human islets and a human β-cell line diminished glucose-stimulated insulin secretion, attenuated Src family tyrosine kinase signaling, and small GTPase Cdc42 activation. We excluded the requirement for the G protein Gαq/11 in TAS1R3 signaling by using the Gαq/11-specific YM-254890 inhibitor in β-cells. Notably, the significant reduction of TAS1R3 mRNA and protein levels in human type 2 diabetes pancreatic islets, which could be replicated in otherwise healthy cells exposed to diabetogenic stimuli, indicates that the TAS1R3 deficit may be a consequence of diabetogenic stimuli.

**Conclusion:**

Overall, our results suggest that TAS1R3 plays an essential role in GTPase signaling in islet β-cells adding to the growing list of proteins that play a vital role in islets as therapeutic targets in type 2 diabetes.

## Highlights

- Human type 2 diabetic pancreatic islets show reduced TAS1R3 levels (>50%). Exposure of healthy cells to diabetogenic stimuli simulates this reduction, indicating that diabetogenic stimuli may cause TAS1R3 deficit.
- TAS1R3 plays an important role in small GTPase signaling in human islets and islet β-cells.
- TAS1R3 is a putative therapeutic target to remediate islet defects associated with type 2 diabetes.

## Introduction

Approximately 98 million US adults have prediabetes (1), which is characterized by fasting hyperglycemia (100–125 mg/dl) or impaired glucose tolerance. Furthermore, 1.5 million US adults annually progress from prediabetes to type 2 diabetes (T2D). The current barriers to preventing prediabetes include unsustainable durable lifestyle interventions (i.e., diet and exercise) and/or inadequate current pharmacological options. Therefore, novel therapies that can prevent and/or reverse prediabetes and T2D are urgently needed.

Normally, elevated circulating glucose increases the glycolytic flux into β-cells, initiating signaling cascades that ultimately lead to Ca^2+^ influx followed by translocation of insulin-laden vesicles toward the plasma membrane for release of their insulin cargo into the circulation (2).

Pancreatic β-cells play a central role in maintaining glucose homeostasis through regulated insulin secretion (3). While glucose metabolism is the primary driver of insulin release, emerging evidence suggests that alternative signaling pathways can modulate this process (4). Notably, the sweet taste receptor (TAS1R), a class C G-protein-coupled receptor (GPCR) heterodimer composed of TAS1R2 and TAS1R3, was identified in β-cell (5) sweet taste receptors, composed of TAS1R2/TAS1R3 heterodimers, which have been identified in human and mouse β-cells (5, 6). Furthermore, fructose synergizes with glucose to enhance insulin secretion in pancreatic β-cells by activating the sweet taste receptor heterodimer TAS1R2/TAS1R3. Taste receptor activation initiates two distinct signaling arms: The Gβγ subunit activates PLCβ2, triggering the IP_3_/Ca²^+^ signaling cascade (7), whereas Gα-gustducin stimulates phosphodiesterases (PDEs) to reduce intracellular cAMP levels (8). In contrast, certain sweeteners may engage Gαs, a distinct Gα subunit, to elevate cAMP, suggesting ligand-specific modulation of intracellular signaling. These parallel pathways such as Ca²^+^ mobilization and cAMP regulation converge to fine-tune cellular responses to tastants. Notably, in pancreatic β-cells, PLCβ2 activation is required for fructose-induced insulin secretion (5), and while cAMP elevation by sugars may involve Gαs, the identity and functional role of Gαs in β-cells remains to be fully elucidated. Interestingly, other GPCRs such as GPR119, a Gαs-coupled receptor, are largely restricted to insulin-producing β-cells (9) and function as glucose-dependent insulinotropic receptors, highlighting the potential for Gαs-mediated pathways in β-cell physiology.

Notably, Gα-gust-knockout mice exhibit significantly impaired glucose homeostasis, both following a glucose challenge and after refeeding post-fasting on standard chow (10). Similarly, saccharin, a common artificial sweetener, can enhance insulin secretion in β-cells by activating TAS1R3 and triggering a PLC-dependent Ca^2+^ signaling pathway (5). Although sweeteners have been reported to induce a delayed increase in cyclic AMP (cAMP) correlating with insulin release, the directness of this effect remains uncertain.

Trimeric GTPases and small Rho family GTPases are known to coordinate and cross-communicate during intracellular signaling cascades (11). However, the specific trimeric GTPases involved in Cdc42-mediated glucose-stimulated insulin secretion (GSIS) from pancreatic β-cells remain unidentified. Recent evidence implicates the sweet taste receptor TAS1R3 in whole-body glucose homeostasis (12), as TAS1R3 knockout mice exhibit impaired glucose tolerance and reduced insulin secretion from MIN6 β-cells (13). Beyond the pancreas, TAS1R3 has also been shown to regulate GLP-1 production in the intestine (14) and amino acid metabolism in skeletal muscle (15), suggesting a broader role in metabolic regulation. Despite these findings, the mechanisms by which TAS1R3 interfaces with small Rho GTPases in β-cells are not yet understood. Therefore, it is critical to investigate the role of TAS1R3 specifically in the context of glucose activation, independent of artificial sweeteners, to address this fundamental gap in our understanding of β-cell signaling and insulin secretion.

In this study, we hypothesized that TAS1R3 is required for GSIS *via* influencing small GTPase Cdc42. To test this hypothesis, we used a combination of pharmacological and molecular approaches in human donor islets and human clonal β-cells. We demonstrated that TAS1R3 is required for GSIS utilizing the small Rho family GTPase Cdc42. Furthermore, T2D human islets showed deficient levels of TAS1R3 mRNA and protein, suggesting that TAS1R3 deficiency may be a consequence of diabetogenic stress.

## Research design and methods

### Human islets

Cadaveric non-diabetic and T2D islets were obtained from the Integrated Islet Distribution Program (IIDP) and from the City of Hope Islet Core (see **Supplementary Table S1**). Upon arrival, human islets were allowed to recover for 2 h in Connaught Medical Research Laboratories-1066 (CMRL-1066; Thermo Fisher, Waltham, MA), 0.61 g niacinamide, 500 μl ITS Premix Universal Culture Supplement (Thermo Fisher, Waltham, MA), 835 μl Zn_2_SO_4_ (10 mM stock), 25 ml sodium pyruvate (100 mM stock), 5 ml GlutaMAX (Thermo Fisher, Waltham, MA), 12.5 ml HEPES (1 M stock), 10% FBS, 5.6 mM glucose, 100 IU/ml penicillin, and 0.1 g/l streptomycin., and then islets were handpicked under a dissecting microscope to yield a purity of >95%.

### Human and rat β-cell culture

EndoC-βH5 and EndoC-βH1 clonal human β-cells were purchased from Human Cell Design (Toulouse, France) and were cultured and used for assays as described in the product manual. INS-1 832/13 cells, gifted from Dr. Christopher Newgard (Duke University Medical Center, Durham, NC), were cultured described previously (16); passage 52–68 were used.

### Quantitative PCR

Total RNA was isolated from human donor samples or INS-1 832/13 cells using the TRI Reagent according to the manufacturer’s protocol (Millipore Sigma, St. Louis, MO, USA) and assessed using two-step reverse transcription (iScript™ cDNA Synthesis Kit, Bio-Rad, Hercules, CA) and qPCR (iQ SYBR® Green Supermix, Bio-Rad, Hercules, CA). Primers used are in **Supplementary Table S2**. The cycle threshold data were converted to change fold in expression by the “δδCt” method.

### TAS1R3 and Gαq/11 inhibition

Human islets, EndoC-βH1, and EndoC-βH5, INS-1 832/13 cells were exposed to 1 mM lactisole that binds to the transmembrane domain of TAS1R3 and requires four key residues for lactisole’s sensitivity (17) (Cayman Chemical Company, Ann Arbor, MI) for TAS1R3 antagonism, or 10 μM YM-254890 for Gαq/11 specific antagonism or 0.1% v/v DMSO (vehicle control), by preventing its conversion to its active GTP-bound conformation (18). Under certain circumstances, INS-1 832/13 cells were exposed to fenofibrate (10 or 27.17 μM); for TAS1R3 inhibition for 20 min.

### Static incubation assay for GSIS

Human islets were incubated for 2 h at 37 °C in Krebs-Ringer bicarbonate buffer (KRBH) as described previously (19). For TAS1R3 or Gαq/11 inhibitor studies, islets were exposed for 20 min (as above), followed by 1 h stimulation with either 1 mM (low glucose) or 20 mM glucose (high glucose). Secreted insulin was measured using an ELISA kit for human insulin (Mercodia, Winston Salem, NC). Insulin content from donor islets solubilized in Nonidet P-40 lysis buffer was quantified by ELISA relative to total islet protein for each batch of human islets using DC Protein Assay kit (Bio-Rad, Hercules, CA).

EndoC-βH5 cells were seeded onto βCoat®-coated 96-well plates. Six days later, medium was replaced with Ulti-ST® starvation medium containing 0.5 mM glucose for 24 h. Medium was then replaced with βKrebs® GSIS buffer supplemented with 0.1% fraction V fatty acid free BSA for 40 min, followed by addition of the TAS1R3 or Gαq/11 inhibitor for 20 min. Cells were then incubated with βKrebs®/BSA supplemented with high glucose (16.7 mM) for 45 min. Incubation medium was collected, spun down, and analyzed by ELISA. For insulin content, cells were lysed in Tris/Triton X-100 based lysis buffer for 5 min, collected, and analyzed by ELISA. Insulin was measured by ELISA using Human Insulin Kit (Mercodia, Winston Salem, NC) following manufacturer’s instructions.

### Glucolipotoxicity exposure

INS-1 832/13 cells were grown in INS-1 medium as described previously (16) until 80% confluent and then cultured in INS-1 medium containing 25 mM glucose and 0.4 mM palmitate (20) for various time periods, as indicated in the figure legends.

### Small interfering RNA transfection

siRNA against TAS1R3 and control non-targeting siRNA duplexes were purchased from OriGene (Rockville, MD). siRNA oligonucleotides were transfected into INS-1 832/13 cells using Lipofectamine™ RNAiMAX transfection reagent (Thermo Fisher, Waltham, MA) and Opti-MEM (Thermo Fisher, Waltham, MA) at 1.0, 10, and 50 nM. Post-36 h transfection, INS-1 832/13 cells were equilibrated in 2.5 mM glucose and 2.5% serum culture medium overnight, followed by a 1 h exposure to glucose-free KRBH. Secreted insulin during GSIS was assayed as above. Post-GSIS assay, cells were harvested in 1% NP-40 lysis buffer to determine TAS1R3 knock-down efficacy by immunoblot.

### Immunoblot analysis

Rabbit polyclonal TAS1R3 (Thermo Fisher Scientific, Waltham, MA) were used to detect the abundance of TAS1R3 from human islets and EndoC-βH1. For active phospho-SFK (Tyr^416^), rabbit polyclonal antibody (Thermo Fisher Scientific, Waltham, MA) was used. As a control for equivalent protein loading in individual wells of the polyacrylamide gel, the content of t-SFK was detected using a rabbit monoclonal antibody (Cell Signaling Technology, Danvers, MA). Goat anti-rabbit (1:10,000, Bio-Rad, Hercules, CA) or goat anti-mouse (1:20,000, Bio-Rad, Hercules, CA) HRP-conjugated secondary antibodies were used for enhanced chemiluminescence detection (ECL, GE Healthcare, Chicago, IL). The complete list of antibodies used in this study is provided in **Supplementary Table S3**.

For the detection of membrane-bound TAS1R3, rabbit polyclonal TAS1R3 (as above) was used. As a control for equivalent protein loading in individual wells of the polyacrylamide gel, the content of STX4 was detected using mouse monoclonal STX4 (BD Transduction Laboratories, San Diego, CA). HRP-conjugated secondary antibodies were used for ECL detection (as above). Mouse monoclonal Rho-GDIα antibody (Santa Cruz Biotechnology, Inc., Dallas, TX), a cytosolic marker, was used to measure the purity of plasma membrane fractions, and whole-cell lysates were used as a positive control.

### Cdc42 and p-SFK(Tyr^416^) activation assay

Human islets or EndoC-βH1 cells were equilibrated in low-glucose (2.5 mM) and low-serum (2.5%) culture medium overnight, followed by a 1-h exposure to glucose-free KRBH. Cells were exposed to KRBH containing antagonists (as above), after which the experiment was conducted for 1 min (phosphorylation-SFK activation) and 2 min (Cdc42 activation) using fresh test solutions with low (2.8 mM) or high (16.7 mM) glucose. After stimulation, cells were harvested in 1% Nonidet P-40 lysis and Phospho-SFK (Tyr^416^) was determined using immunoblot analysis (see above). G-LISA (Cytoskeleton Inc. (Cat BK 127) was used to quantify the amount of active Cdc42-GTP from human islets and EndoC-βH1 cell lysate as described previously (19).

### Intracellular cAMP measurements

EndoC-βH5 were equilibrated in βKrebs® GSIS buffer supplemented with 0.1% fraction V fatty acid free BSA for 40 min, followed by addition of TAS1R3 or Gαq/11 inhibitor for 20 min. Cells were then incubated with βKrebs®/BSA supplemented with glucose (16.7 mM) for 45 min. On the day of cAMP assay, 1 mM phenylmethylsulfonyl fluoride (PMSF) was added to cell lysates; intracellular cAMP levels were measured using a Direct cAMP ELISA kit from Enzo Life Sciences, Inc. (Farmingdale, NY), as per manufacturer’s protocol.

### Plasma membrane isolation

EndoC-βH1 was equilibrated in low-glucose (2.8 mM) and low-serum (2.5%) culture medium overnight, followed by a 1-h exposure to glucose-free KRBH. Plasma membrane fractions of EndoC-βH1 glucose-stimulated cells (16.7 mM glucose, 15 min). Cells were isolated as previously described (21). The proteins were further separated using SDS-PAGE and transferred to the PVDF membrane. Rho-GDIα, a cytosolic marker, was used to validate the purity of plasma membrane fractions; whole-cell detergent clarified lysates served as positive control for antibody detection capability.

### Statistics

Data are presented as mean ± SEM, and *n* values are indicated in the figures. Due to inherent variability in basal expression and activity levels across different cell lines and experimental replicates, we opted to calculate relative changes using fold-change normalization to each experiment’s internal control. This method of data presentation aligns with approaches used in our previous publications (19, 21). Differences between two groups were assessed using Student’s t-test. Statistically significant differences among multiple groups were evaluated using one-way or two-way ANOVA followed by Bonferroni *post-hoc* test. The threshold for statistical significance was set at p < 0.05 and all analyzed using GraphPad Prism software, version 8.3.0. Statistical significance is indicated in the figure legends.

### Data and resource availability

The data sets generated during and/or analyzed during the current study are available from the corresponding author upon reasonable request. No applicable resources were generated during this study.

## Results

### TAS1R3 mRNA and protein levels are reduced in T2D human islets

**Figure 1:**
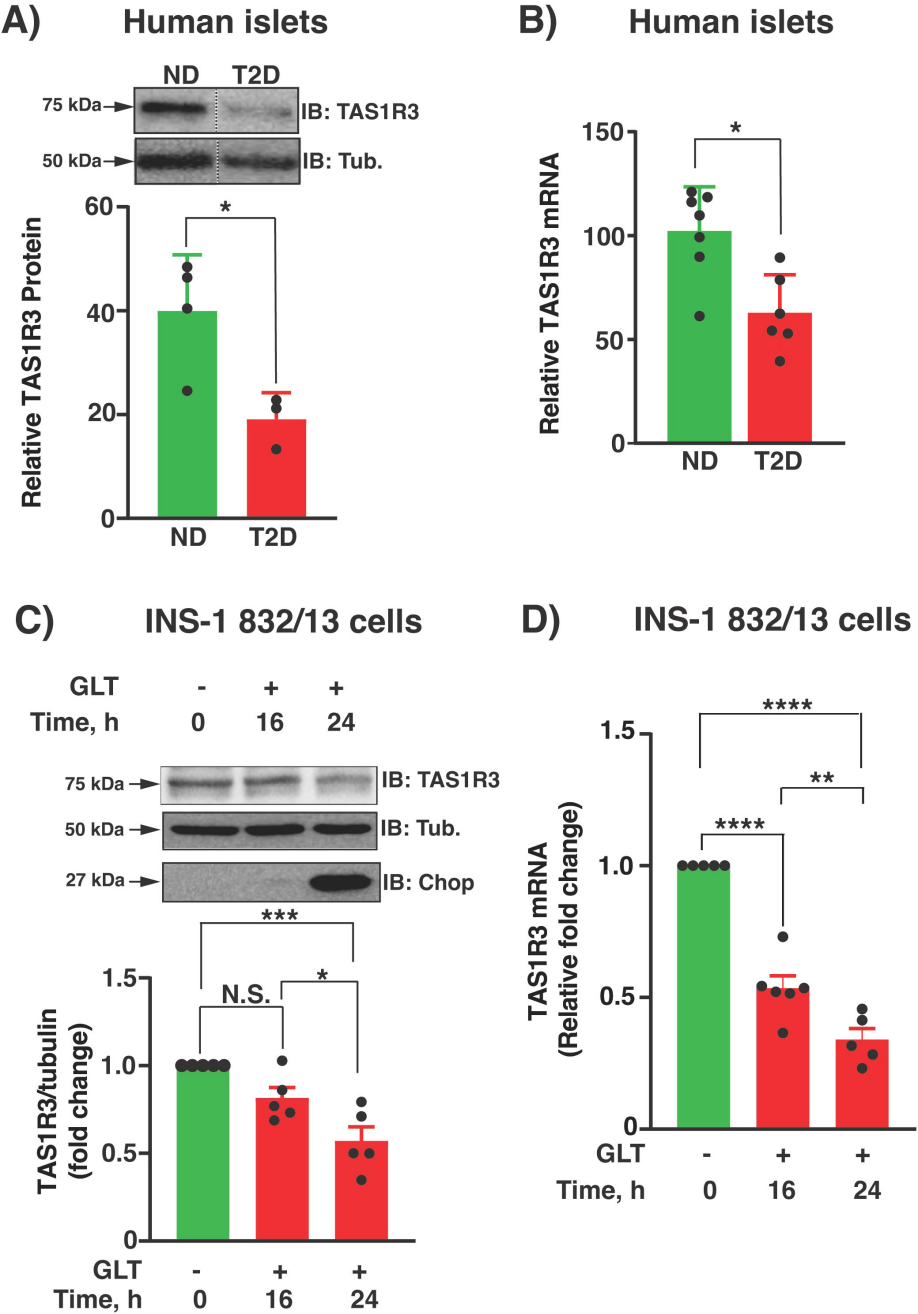
TAS1R3 expression is significantly lower in human islets from T2D individuals compared with non-diabetic individuals. **(A)** Representative immunoblot (IB) is shown. Densitometry analysis of TAS1R3 protein abundance in human T2D (n=3 donors) versus non-diabetic (ND; n=4 donors) islets. Top: Vertical dashed line indicates splicing of lanes from within the same gel exposure. Control: tubulin (Tub). **(B)** Quantification of TAS1R3 mRNA expression relative to tubulin mRNA expression in human T2D (n=6 donors) versus ND (n=7 donors) islets by qPCR. **(C, D)** INS-1 832/13 cells were untreated (0 h) or exposed to glucolipotoxic (GLT) stress (0.4 mM palmitate and 25 mM high glucose) for up to 24 h (n=5 independent experiments). **(C)** Representative immunoblot (IB) is shown. Densitometry TAS1R3 protein abundance relative to tubulin protein levels. Top: Representative IB. CHOP indicates ER stress induction with GLT treatment. **(D)** Quantification of TAS1R3 mRNA (normalized to tubulin) by qPCR. TAS1R3 mRNA at 0 h (fold-change set to 1.0) compared with 16 and 24 h of exposure. **(A–D)** Data expressed as mean ± SEM; N.S., not significant. *p < 0.05, **p < 0.01, ***p < 0.001. ****p < 0.0001.

TAS1R3 abundance was significantly reduced in T2D islets compared with non-diabetic controls (**Figures 1A, B**). Furthermore, a time-dependent reduction in TAS1R3 was confirmed in rat clonal β-cells (INS-1 832/13) exposed for 16 or 24 h to diabetogenic glucolipotoxic (GLT: 25 mM glucose, 400 μM palmitic acid) stress (**Figures 1C, D**). The concentrations of GLT used in the current studies have been validated in INS-1 832/13 clonal β-cells (20, 22). Given that endoplasmic reticulum (ER) stress is a hallmark feature of GLT-induced β-cell dysfunction, we measured CHOP. CHOP levels were significantly elevated within 24 h of GLT exposure (**Figure 1C**). This suggests that the reduction in β-cell TAS1R3 levels may result from glucolipotoxic diabetogenic stress in T2D human islets.

### Inhibition of TAS1R3, and its potential downstream G-protein target Gαq/11, impairs GSIS, but only TAS1R3 is required for cAMP

**Figure 2:**
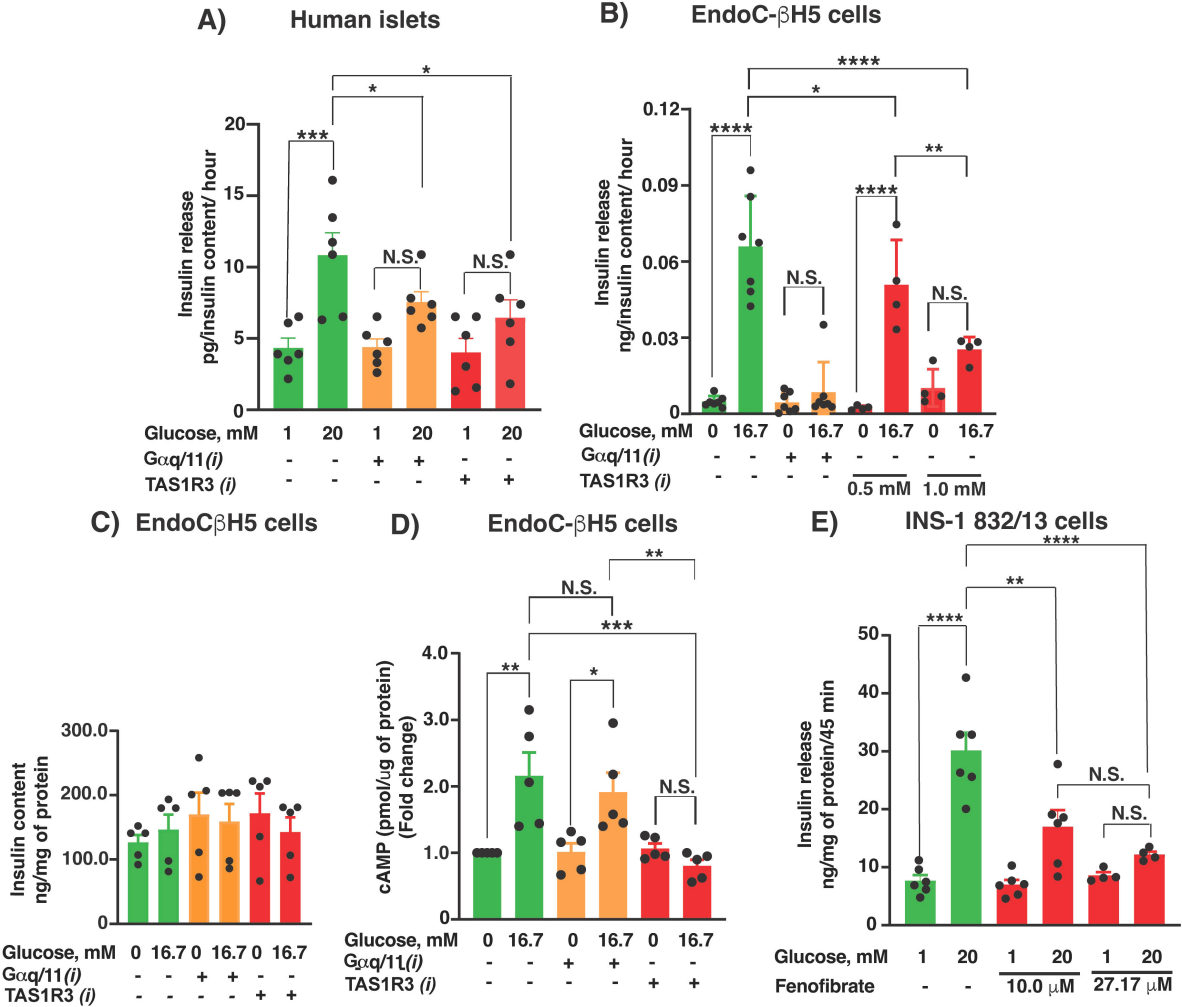
Lactisole and fenofibrate inhibit GSIS from human islets and human EndoC- βH5 cells. Human islets **(A)**, n=6 islet donors) and EndoC-βH5 **(B)**, 4–7 independent experiments) were incubated with TAS1R3 inhibitor (lactisole, 1 mM) or Gαq/11 inhibitor (YM-254890, 10 μM) to test their abilities to alter GSIS. Vehicle: DMSO. The indicated compounds were added for 20 min prior to stimulatory glucose exposure (20 mM for 1 h in **(A)** or 16.7 mM for 45 min in **(B)**. Secreted insulin values were normalized to the insulin content. **(C)** EndoC-βH5 cells were preincubated with test compounds for 20 min and then exposed to stimulatory glucose (16.7 mM) for 45 min (n=5 independent experiments). cAMP levels were quantified and normalized to total protein. **(D)** Effect of cholesterol drug and TAS1R3 inhibitor, fenofibrate (10 and 27.17 μM), on INS-1 832/13 cell GSIS. Fenofibrate was added 20 min pre-stimulatory glucose (20 mM) exposure. N = 6 independent experiments. Secreted insulin values were normalized to total cellular protein content. **(A-D)** Data expressed as mean ± SEM; N.S., not significant. *p < 0.05, **p < 0.01, ***p < 0.001. ****p < 0.0001.

TAS1R3 is known to interact with multiple G-protein subfamilies (23, 24). Furthermore, β-cell–specific deletion of Gαq and Gα11 impairs glucose tolerance and insulin secretion due to loss of muscarinic or metabolic potentiation, and a diminished response to glucose itself (25). CaSR, a Gαq/11-coupled receptor, is activated by kokumi substances (e.g., γ-glutamyl peptides) and enhances sweet, salty, and umami taste perception (26) using some of the downstream components of the canonical TAS1R3 pathway (24). Therefore, we used YM-254890, a selective Gαq/11 inhibitor (18), to test the hypothesis that Gαq/11 contributes to TAS1R3-driven insulin secretion and influences cAMP and small GTPases. We assessed GSIS inhibition in non-diabetic human islets (**Figure 2A**) and human EndoC-βH5 cells (**Figure 2B**) following selective inhibition of Gαq/11 (YM-254890, Gαq/11*i*) or TAS1R3 (lactisole, TAS1R3*i*). The concentration (10 μM) we used in our current study for YM-254890, a selective inhibitor of Gαq/11 (13), had a minimal effect on the sucralose-induced elevations in intracellular calcium Ca^2+^ and cAMP. In contrast, YM-254890 completely abolished the increases in Ca^2+^ and cAMP elicited by carbachol, a known Gq/11-coupled receptor agonist in MIN6 cells (27). Both inhibitors significantly reduced GSIS compared with the vehicle control. TAS1R3*i* exhibited a dose-dependent inhibition of GSIS in the EndoC-βH5 cells (**Figure 2B**, bar 8 vs. bar 6); therefore, the higher dose (1 mM) was used for all subsequent studies. Importantly, the observed unaltered insulin content following TAS1R3 inhibition (**Figure 2C**) was consistent across both pharmacological agent tested, reinforcing the specificity of the effect and minimizing the likelihood that pH alterations were responsible. Previously, cAMP was shown to be a critical metabolic coupling factor for GSIS by acting through Epac2 to activate the Cdc42-Pak1 pathway and facilitate insulin granule mobilization (19). Inhibition of cAMP signaling blocks GSIS and cytoskeletal remodeling, whereas Epac activation restores insulin secretion even in T2D islets (19, 28, 29). Similarly, Saccharin, a common artificial sweetener, can enhance insulin secretion in β-cells by activating TAS1R3 and triggering a PLC-dependent Ca^2+^signaling pathway (5). Although sweeteners have been reported to induce a delayed increase in cyclic AMP (cAMP) correlating with insulin release, the directness of this effect remains uncertain.

Therefore, we measured cAMP levels post-Gαq/11 or -TAS1R3 inhibition in EndoC-βH5 cells. As expected, stimulatory glucose evoked a significant increase in cAMP levels in vehicle-treated cells; on the other hand, cAMP levels were only reduced post-TAS1R3 inhibition, whereas Gαq/11 inhibition showed no effect (**Figure 2D**).

We next tested the cholesterol-lowering drug fenofibrate, which is used in T2D. Based on a T2D patient case report, fenofibrate can impair sweet taste perception; this effect is reversed upon drug discontinuation and recurs upon rechallenge (30). Thus, fenofibrate may inhibit human TAS1R3. Indeed, long-term fenofibrate treatment impairs GSIS in obese rats (31). Consistent with findings from a previous study (32), fenofibrate at 100 µM reduced KATP channel current in MIN6 β-cells without affecting insulin mRNA expression. Notably, the concentrations used in that study were significantly higher than those employed in the current study (10 and 27.17 µM), reducing the likelihood that our observed effects are due to off-target mechanisms (**Figure 2E**). The fenofibrate dose of 27.17 μM, as used in this study, is clinically relevant in humans, since a single dose of 300 mg fenofibrate reaches concentrations of approximately 10 mg/L in plasma, which is 27.17 μM (33).

**Figure 3:**
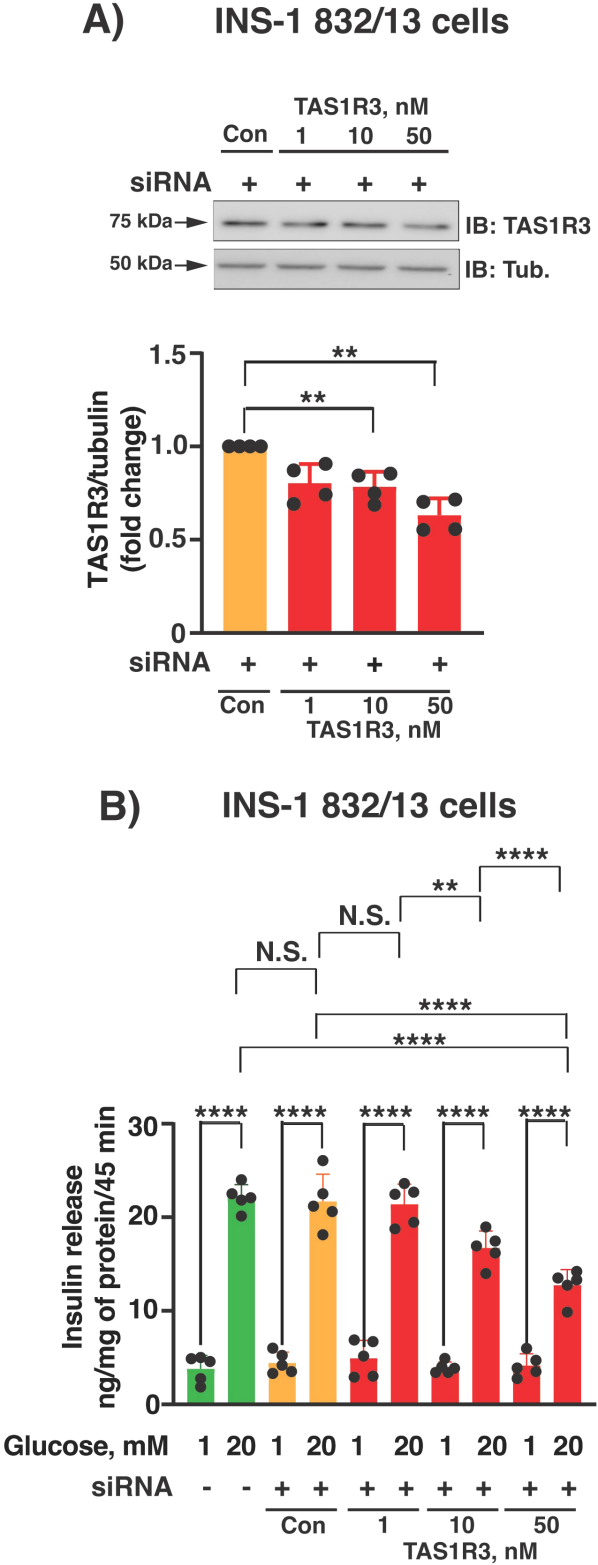
TAS1R3 knockdown attenuates GSIS from rat INS-1 832/13 cells. **(A)** Impact of TAS1R3 knockdown with small interfering RNA (siRNA) on TAS1R3 protein abundance in INS-1 832/13 cells. Top: Representative immunoblot (IB) is shown. Bottom: densitometry analysis of TAS1R3 protein expression normalized to tubulin from siRNA-treated cells (n=4 independent experiments). TAS1R3 abundance from non-transfected cells was set to 1.0. **(B)** Impact of TAS1R3 siRNA knockdown on GSIS in INS-1 832/13 cells. Post-siRNA treatment, cells were exposed to glucose (20 mM) for 45 min (n= 5 independent experiments). Secreted insulin values were normalized to total cellular protein content. **(A, B)** Data expressed as mean ± SEM; N.S., not significant.**p < 0.01 and ****p < 0.0001.

We further tested the requirement for TAS1R3 in GSIS using siRNA-mediated knockdown. In INS-1 832/13 cells, we observed a concentration-dependent knockdown of TAS1R3 (0–50 nM), achieving ~45% knockdown efficiency using 50 nM of TAS1R3-specific-siRNA as compared with control-siRNA at the protein level (**Figure 3A**). TAS1R3-siRNA dose-dependently inhibited GSIS compared with control siRNA or non-transfected cells (**Figure 3B**). Overall, both pharmacological inhibition and genetic knockdown of TAS1R3 suggests that it is required for GSIS.

### Inhibition of TAS1R3 attenuates Src family kinase and Cdc42 activation events required for GSIS

**Figure 4:**
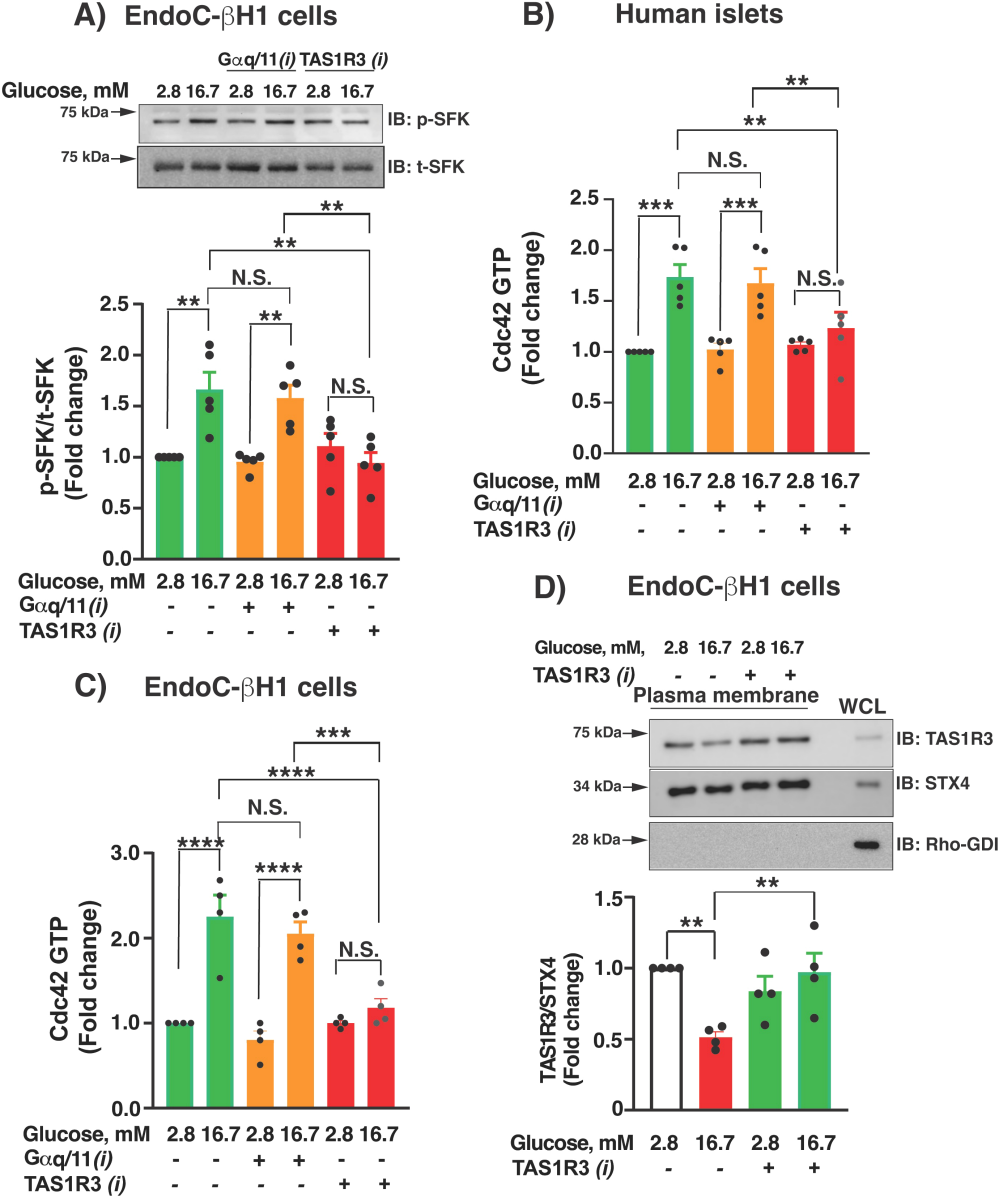
Lactisole inhibits whereas YM-254890 fails to inhibit phospho-SFK, and Cdc42 activation in EndoC-βH1cells and human islets attenuates plasma membrane-localized TAS1R3 abundance. EndoC-βH1 cells **(A, C)** and human islets **(B)** were treated with TAS1R3*i* lactisole (1 mM) or Gαq/11*i* YM-254890 (10 μM) for 20 min. Following this, cells were exposed to glucose (2.8 or 16.7 mM) for 1 min for phospho-SFK (p-SFK) activation **(A)** and 2 min for Cdc42 activation **(B, C)**. **(A)** Representative immunoblot (IB) is shown. Densitometry analysis of p-SFK normalized to t-SFK (n=5 independent experiments). **(B, C)** Cdc42-GTP was detected by G-LISA (n=5 donors in **(B)** and n=4 independent experiments in **(C)**. Values are relative to baseline Cdc42-GTP levels detected with 2.8 mM glucose stimulation. **(D)** Plasma membrane fractions were isolated from 2.8 and 16.7 mM glucose-stimulated EndoC-βH1 cells and treated with TAS1R3*i* lactisole (15 min). Top: Representative immunoblot (IB) is shown. Rho-GDI, a cytosolic marker, demonstrates purity of the PM fractions. For densitometry analysis, abundance of TAS1R3 was normalized relative to syntaxin 4 (STX4) levels (n=4 independent experiment). WCL: whole-cell lysates. Data expressed as mean ± SEM; N.S., not significant. **p < 0.01, ***p < 0.001. ****p < 0.0001.

The glucose-stimulated activation of Cdc42 in β-cells requires upstream YES kinase (an Src family kinase, SFK) signaling (34). To date, YES kinase is the only member of the SFK family that responds to high glucose in β-cells, detected using a pan-SFK pTyr416 antibody. Since glucose-stimulated cAMP accumulation enhances Cdc42 activity (19), we questioned if TAS1R3 played a role in SFK-mediated Cdc42 activation. We treated EndoC-βH1 cells with TAS1R3*i* or Gαq/11*i* inhibitor and measured SFK activation and determined that TAS1R3*i* but not Gαq/11*i* blunted pSFK activation (**Figure 4A**). We further assessed the effects of these inhibitors upon Cdc42 activation in human islets and human EndoC-βH1 cells. Compared with vehicle control, only TAS1R3 inhibition significantly ablated Cdc42 activation; Gαq/11 inhibition was without effect (**Figures 4B, C**). Hence, SFK and Cdc42 activation events are TAS1R3-dependent, whereas the Gαq/11 requirement in GSIS occurs *via* a different pathway.

Following activation, most GPCRs employ the β-arrestin-dependent clathrin-mediated endocytosis pathway for receptor desensitization, internalization, and subsequent recycling or degradation (35). As such, we investigated TAS1R3 abundance at the plasma membrane (PM) in human EndoC-βH1 cells after 15 min of stimulatory glucose. Cells were stimulated with high glucose (16.7 mM) or low glucose (2.8 mM) for 15 min and immediately subfractionated to evaluate changes in membrane-associated TAS1R3 abundances. PM-TAS1R3 was significantly diminished post-15 min glucose stimulation, relative to the static plasma membrane protein STX4, compared with TAS1R3 abundance at the PM in unstimulated (2.8 mM glucose) cells (**Figure 4D**). Furthermore, treatment with the TAS1R3 inhibitor lactisole prevented the high-glucose induced reduction of TAS1R3 abundance at the PM at 15 min post-glucose stimulation. Consistent with other glucose-sensitive functional GPCR in β-cells (36), these findings suggest that TAS1R3 internalizes in response to stimulatory glucose.

## Discussion

**Figure 5:**
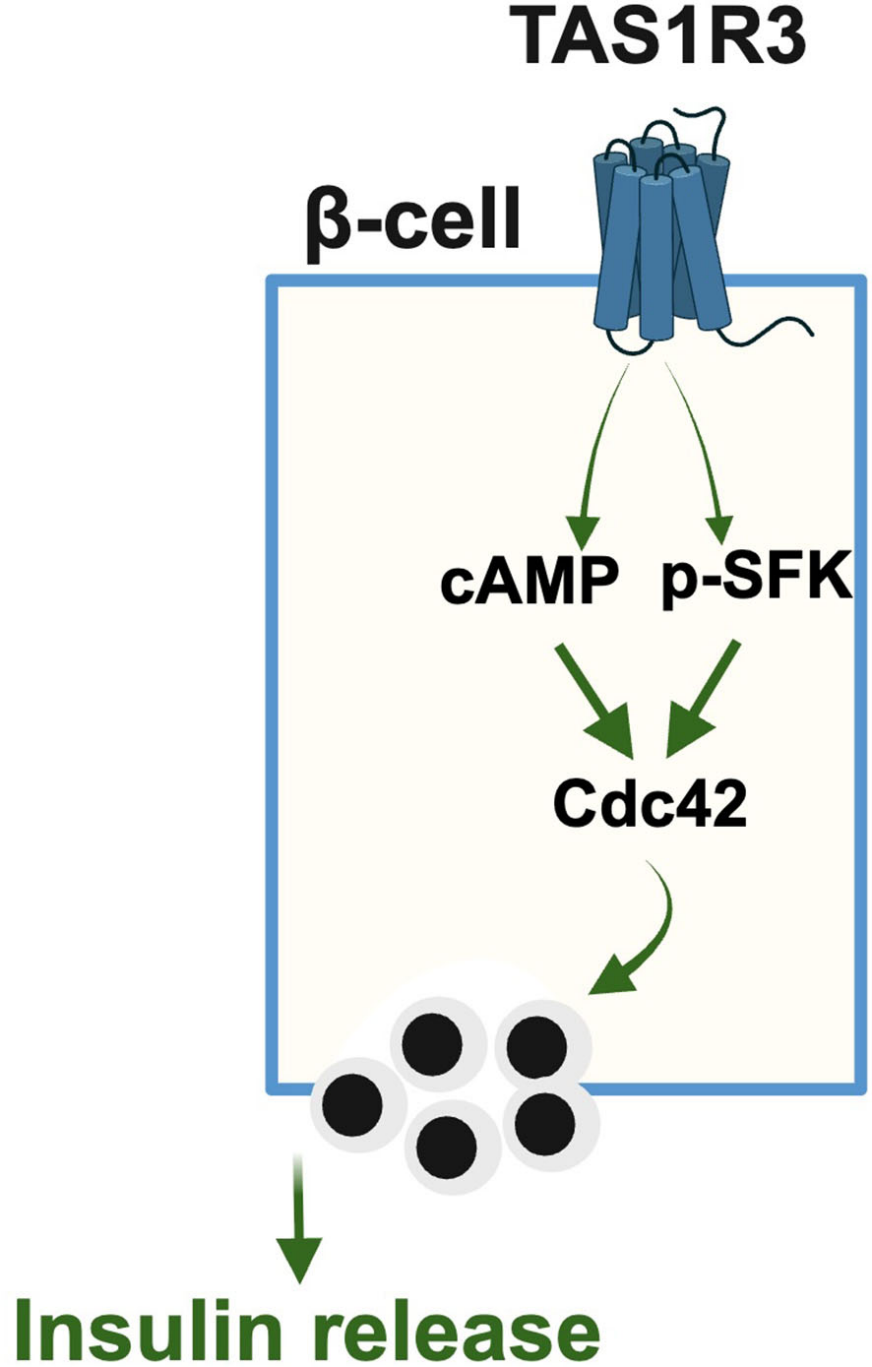
Activation of TAS1R3 elicits two convergent pathways: generation of cAMP and phosphorylation of Src family kinases (p-SFK). These signals culminate in Cdc42 activation, driving F-actin remodeling to facilitate insulin granule exocytosis.

This study provides novel mechanistic insights into the glucose-stimulated role of TAS1R3-mediated trimeric GTPase signaling events that facilitate GSIS from β-cells. There are two SNPs associated with TAS1R3 genes (TAS1R3 rs307355 and rs35744813) and prior multivariate analysis normalized for age, sex, and BMI showed a significant decrease in sensitivity to sweet stimuli, suggesting that TAS1R3-TAS1R2 is important for sweet sensing (37). Here, we showed that human T2D islets displayed significantly reduced TAS1R3 levels compared with non-diabetic controls, consistent with the impaired glucose tolerance and insulin resistance phenotype observed in the TAS1R3 knockout mice (12). Furthermore, we revealed a β-cell specific role for TAS1R3 in non-canonical Cdc42 signaling *via* TAS1R3 antagonism. Collectively, our findings emphasize TAS1R3 as a promising dual-function metabolic regulator, with roles in both sweet taste perception and β-cell signaling (**Figure 5**). The observed reduction of TAS1R3 in T2D islets, along with its involvement in non-canonical Cdc42 signaling highlights its potential as a therapeutic target.

These findings elucidate the most upstream event, TAS1R3 activation, spearheading a non-canonical signaling cascades required for GSIS from β-cells. TAS1R3 did not influence Gαq/11 and instead influences *via* the Rho family GTPases-Cdc42 in β-cells, which is required for actin remodeling *via* PAK1 activation (38–40). In β-cells, PAK1 signaling diverges through Rac1 and Raf/MEK/ERK to culminate in a net decrease in F-actin at the cortical plasma membrane (41, 42). Currently, GPCR ligands/agonists are being pursued in a therapeutic context given the ability to activate them extracellularly and harness their signaling cascades to evoke insulin secretion.

Our use of selective short-duration pharmacologic inhibitors of TAS1R3 activity supports the concept that there is an acute need for TAS1R3 signaling in β-cells. Moreover, our GLT results suggested TAS1R3 transcriptional and post-transcriptional downregulation, supporting the concept that exposure to dietary diabetogenic stimuli causes loss of TAS1R3 in β-cells. We further corroborated this reduction in TAS1R3 mRNA and protein levels in human T2D islets. Additionally, we found that fenofibrate inhibited GSIS *in vitro*. Fenofibrate is a commercially available drug used to treat clinical dyslipidemia (43). A structurally related compound from the fibrate family, clofibrate, now discontinued from the market, has been identified as a known inhibitor of the sweet taste receptor TAS1R3 (44). Notably, fenofibrate also exerts an inhibitory effect on human TAS1R3 (30), although direct molecular evidence supporting this mechanism is currently lacking. Yet, in spite of the beneficial effect on plasma lipid levels (plasma triacylglycerol and NEFA), fenofibrate treatment (0.1%; 3 weeks) of mice almost completely abolished the acute phase of insulin secretion (43),. Our findings are consistent with this evidence and contribute to the mechanistic understanding of the observed diabetes risk with fenofibrate use.

TAS1R3 activation precedes Src kinase YES activation, revealing the foremost signaling event triggering the signaling cascade linking small GTPases→actin remodeling→GSIS in β-cells. The glucose-stimulated phosphorylation of SFK, particularly of YES kinase, was previously reported to be a key proximal step in the activation of Cdc42 (34), and essential for actin cytoskeletal remodeling. Lactisole antagonism of TAS1R3 blunted glucose induced activation of phospho-SFK and of Cdc42, suggesting a non-canonical signaling cascade emanating from TAS1R3 signaling in the β-cell. The binding of Gα subunits of trimeric GTPases to the SFK catalytic domain causes conformational changes in SFK family members, leading to increased accessibility of the active site to substrates (45) and, in this case, Cdc42. The activation of SFK by trimeric GTPases is specific for Gα and Gαi, but not for Gαq, Gα12, or Gβy (45). Yet, another possible mechanism involves β-arrestin acting as multifunctional scaffold proteins that, upon coupling to activated GPCRs, initiate alternative signaling cascades either independently of or in conjunction with G-proteins. Through these interactions, β-arrestin can recruit and organize signaling complexes involving MAPK components and nonreceptor tyrosine kinases such as Src, thereby modulating diverse cellular processes including chemotaxis, apoptosis (46), and potentially β-cell activation of Cdc42. While it is true that multiple GPCRs, including CaSR, can converge on Gαq/11-mediated signaling pathways and insulin secretion (25, 47), the specificity of YM-254890 for Gαq/11 has been well characterized (18). As demonstrated in HL60, CHO and MIN6 cell (27) models YM-254890 at 10 μM selectively inhibits Gαq/11-mediated PLCβ activation and downstream Ca²^+^ mobilization, without affecting Gαi-or Gα15-mediated signaling, nor does it interfere with downstream effectors such as PLCβ, IP3 receptors, or store-operated calcium channels. In our study, the use of YM-254890 allowed us to specifically interrogate the role of TAS1R3 versus Gαq/11 in GSIS and downstream activation of Cdc42. While our data support the conclusion that Gαq/11 likely exerts an independent effect from TAS1R3 activation. However, we cannot fully exclude the possibility that synergistic activation of Gαq/11-coupled receptors, such as CaSR, may contribute to the induction of GSIS. Gq likely exerts an independent effect from TAS1R3 activation. Further studies are required to identify the trimeric GTPase cascade that potentially occur concurrently, yet independently of TAS1R3 signaling, to regulate GSIS.

One potential limitation in the current study is the lack of demonstration of TAS1R3 functional implications in cell type-specific conditional knockout animal models *in vivo*; at present, only classic whole-body models exist, and given the importance of TAS1R3 in multiple endocrine cell types that contribute to glucose homeostasis, future studies will require generation of inducible, conditional knockout mouse models. These emerging mechanistic insights facilitate the addition of TAS1R3 to the growing list of factors that play a role in β-cells as a therapeutic target that remediate islet defects associated with T2D.

## Data Availability

The original contributions presented in the study are included in the article/**Supplementary Material**. Further inquiries can be directed to the corresponding author.
