# Supplementary material for: TAS1R3 influences GTPase-dependent signaling in human islet β-cells
Source: Front Endocrinol (Lausanne). 2025 Dec 8;16:1695980. doi: 10.3389/fendo.2025.1695980 (PMC12719281; doi:10.3389/fendo.2025.1695980)
Supplement: Supplementary file 1 [file Table1.docx]

**SUPPLEMENTAL MATERIAL**

**TAS1R3 influences GTPase dependent signaling in human islet β-cells**

Rajakrishnan Veluthakal^*^, Miwon Ahn, Eunjin Oh and Debbie C. Thurmond

Department of Molecular & Cellular Endocrinology, Arthur Riggs Diabetes and Metabolism Research Institute of City of Hope, Duarte CA.

^*^Corresponding author

Rajakrishnan Veluthakal, Ph.D

[rveluthakal@coh.org](mailto:rveluthakal@coh.org)

**Short title:** *TAS1R3 functions in human islets*

**Table of Contents**

**Supplemental Table 1 …………………………………………………………. S2**

**Supplemental Table 2 …………………………………………………………. S3**

**Supplemental Table 3 …………………………………………………………. S4**

| **RR1D** | **Sex** | **Age(Y)** | **BMI** | **Race** | **Islet Purity (%)** | **Islet Viability(%)** | **Exptl use** | **HbA1c** | **Type of islets** |
| --- | --- | --- | --- | --- | --- | --- | --- | --- | --- |
| SAMN10832912 | M | 54 | 33.7 | African/American | 90 | 94 | qPCR TAS1R3, WB | 8.0 | T2D |
| SAMN10953189 | F | 56 | 23.1 | Caucasian | 90 | 95 | qPCR TAS1R3, WB | 7.2 | T2D |
| SAMN08774473 | M | 51 | 35.8 | Hispanic | 80 | 90 | qPCR TAS1R3 | 5.3 | T2D |
| SAMN10715692 | M | 63 | 29.5 | Others | 80 | 95 | qPCR TAS1R3, | 11.9 | T2D |
| SAMN28157682 | M | 37 | 30.3 | Caucasian | 80 | 96 | qPCR TAS1R3 | 5.0 | ND |
| SAMN28156887 | M | 29 | 22.2 | Hispanic | 80 | 95 | qPCR TAS1R3 | 5.6 | ND |
| SAMN15337453 | M | 26 | 24.2 | Hispanic | 98 | 95 | qPCR TAS1R3 | 5.2 | ND |
| SAMN13570019 | F | 37 | 24.0 | Caucasian | 95 | 96 | qPCR TAS1R3, WB | 5.2 | ND |
| SAMN11982795 | M | 54 | 42.8 | Caucasian | 90 | 98 | qPCR TAS1R3 | 6.9 | ND |
| SAMN114833342 | F | 52 | 39.8 | Caucasian | 92 | 98 | GSIS, WB | 6.4 | ND |
| SAMN11514696 | F | 59 | 21.8 | African/American | 88 | 90 | GSIS, WB | 5.2 | ND |
| SAMN12500521 | M | 52 | 29.0 | Caucasian | 95 | 98 | GSIS, WB | 5.1 | ND |
| SAMN13320559 | M | 52 | 24.7 | Hispanic | 85 | 95 | GSIS | 4.8 | ND |
| SAMN13027809 | F | 43 | 31.6 | Caucasian | 90 | 95 | GSIS | 5.7 | ND |
| SAMN12924398 | M | 38 | 28.0 | Hispanic | 82 | 95 | GSIS | 5.4 | ND |
| Hu1161-COH | F | 58 | 34.7 | Caucasian | 80 | 99 | qPCR TAS1R3, WB | 10.1 | T2D |
| Hu 986-COH | M | 47 | 26.2 | Hispanic | 75 | 93.5 | qPCR TAS1R3 | 7.5 | T2D |
| Hu 1155-COH | M | 52 | 27.2 | Hispanic | 85 | 90.4 | qPCR TAS1R3 | 5.7 | ND |
| Hu 1113-COH | M | 38 | 23.8 | Hispanic | 83 | 92 | qPCR TAS1R3 | 5.1 | ND |
| SAMN43777471 | M | 57 | 28.8 | Caucasian | 95 | 95 | GLISA | 5.9 | ND |
| SAMN43548064 | M | 44 | 29.8 | Caucasian | 90 | 93 | GLISA | 5.4 | ND |
| Hu 1299-COH | M | 54 | 22.3 | Hispanic | 93 | 94 | GLISA | 5.3 | ND |
| Hu 1300-COH | F | 49 | 37.5 | Caucasian | 80 | 88 | GLISA | 5.6 | ND |
| Hu 1302-COH | F | 64 | 23.3 | Caucasian | 90 | 100 | GLISA | 5.0 | ND |

**Supplemental Table 1: Human islet donor profiles.**

Exptl, experimental; ND, non-diabetic; GSIS, glucose stimulated insulin secretion; WB, Western blot; GLISA, Small GTPase activation assay; T2D, type 2 diabetes.

**Supplemental Table 2: Primer sequences used for qRT-PCR.**

| **Gene** | **Species** | **Primer forward (5’ to 3’)** | **Primer reverse (5’ to 3’)** |
| --- | --- | --- | --- |
| TAS1R3 | Human | ACACTCTTCAGTGCAACGCCTC | ACTCCATGTCCACGTTTCCGCT |
| Tubulin | Human | AATGTCGGCCACCTTCATTG | CTCAGCCTCAGTGAACTCCA |
| TAS1R3 | Rat | AACTACACACAGTACCAACCC | GTCACTTAGCCGATCCATGC |
| GAPDH | Rat | AGTTCAACGGCACAGTCAAG | TACTCAGCACCAGCATCACC |

**Supplemental Table 3: Antibodies used for Westerns**

| **#** | **Antibody** | **Cat #** | **Vendor** | **Dilution** |
| --- | --- | --- | --- | --- |
| 1 | Rabbit TAS1R3 Polyclonal Antibody | PA5-102260 | Invitrogen | 1: 1000 |
| 2 | Rabbit Phospho-Src (Tyr^416^) Polyclonal Antibody | PA5-97366 | Invitrogen | 1: 1000 |
| 4 | Rabbit Src Antibody | 2108 | Cell Signaling | 1: 1000 |
| 5 | Mouse Anti-Syntaxin 4 | 610439 | BD Transduction Laboratories™ | 1: 5000 |
| 6 | Mouse Rho GDIα Antibody (G-2) | Santa Cruz Biotechnology | sc-373883 | 1: 5000 |
| 7 | Mouse anti-Tubulin | Millipore Sigma | T5168 | 1: 7500 |
